# A Case of Simultaneous, Biopsy-Proven, Classic, ANCA-Positive Wegener's Granulomatosis and Anti-GBM Disease, but without Detectible Circulating Anti-GBM Antibodies

**DOI:** 10.1100/tsw.2010.107

**Published:** 2010-06-15

**Authors:** Aleksandra Gmurczyk, Shubhada N. Ahya, Robert Goldschmidt, George Kim, L. Tammy Ho, Kevin Nash

**Affiliations:** Evanston Northwestern Healthcare, Northwestern University Feinberg School of Medicine, Chicago, USA

**Keywords:** Wegener’s granulomatosis, anti-GBM antibody, ANCA, kidney injury

## Abstract

Wegener's granulomatosis (WG) is a systemic, necrotizing, granulomatous vasculitis of unknown etiology. Approximately 75% of cases present as classic WG with both pulmonary and renal involvement, while the remaining 25% of patients present with a limited form with either predominantly upper or lower respiratory tract symptoms. Ninety percent of WG patients have circulating anti–neutrophil cytoplasmic antibodies (ANCA), and approximately 10% have both circulating ANCA antibodies and concomitant anti–glomerular basement membrane (anti-GBM) disease on renal biopsy. Virtually all of these patients also have circulating anti-GBM antibodies. While it has been reported that some patients with ANCA vasculitis have circulating anti-GBM antibodies, and patients with anti-GBM disease may have positive ANCA, review of the literature does not demonstrate other cases of biopsy-proven, simultaneous, ANCA-associated vasculitis and anti-GBM disease. We report a case of simultaneous, biopsy-proven, classic, ANCA-positive WG and anti-GBM disease, but without detectible circulating anti-GBM antibodies. We present findings characteristic of both WG and linear IgG deposition along the GBM suggesting concurrent anti-GBM disease, in the absence of detectable circulating anti-GBM antibodies. Possible theories to explain the absence of these antibodies are discussed.

## INTRODUCTION

Wegener's granulomatosis (WG) is a systemic, necrotizing, granulomatous vasculitis of medium and small vessels that can affect both the upper or lower respiratory tract as well as the kidneys. Anti–glomerular basement membrane (anti-GBM) disease is a disorder involving damage of the glomerular and/or alveolar basement membrane that can lead to a crescentic glomerulonephritis and/or pulmonary hemorrhage. Both should be included in the differential diagnosis of rapidly progressive glomerulonephritis and pulmonary-renal syndrome.

While there are no absolute clinical diagnostic criteria, both diseases should be suspected in the setting of acute kidney injury with a nephritic urinary sediment and pulmonary involvement. Laboratory testing can help discern between these two disorders, as well as other possible causes of the pulmonary-renal syndrome. Approximately 90% of patients with active WG have anti–neutrophil cytoplasmic antibody (ANCA) positivity and of these, 80–90% are proteinase-3 (PR-3) positive[1]. Depending on the particular enzyme-linked immunosorbent serologic assay (ELISA), the sensitivity of anti-GBM antibody testing can be between 60 and 100% for anti-GBM disease[2].

However, there is known overlap in serological data between ANCA and anti-GBM antibodies. Studies reveal that between 7.5 and 14% of ANCA-positive sera also have anti-GBM antibodies, and up to 30–38% of those with anti-GBM antibodies have concurrent ANCA[3,4,5,6].

It is unclear whether dual positivity is a combination of both diseases or an epiphenomenon. It is impossible to ascertain based on serological testing alone which of these is clinically present. A biopsy of the kidney and/or respiratory tract tissue is the gold standard for differentiating WG and anti-GBM disease. We present a case of an elderly woman with clinical and pathologic evidence of both WG and anti-GBM disease, in whom serological testing revealed the presence of c-ANCA (cytoplasmic-ANCA) and PR-3, but not anti-GBM antibodies by ELISA.

## CASE REPORT

A 71-year-old, Caucasian woman with a history of recurrent sinusitis, bilateral hearing loss, breast cancer treated with lumpectomy and radiation 14 years ago, hypertension, and stage III chronic kidney disease (GFR 35 mL/min per MDRD equation) presented with 1 week of malaise, nausea, vomiting, diarrhea, and confusion. She also noted chills, productive cough, and decreased urine output for several days. She did not report any hemoptysis. Outpatient medications included chlordiazepoxide/clidinium, thyroxine, ranitidine, risedronate, alprazolam, ramipril, mirtazapine, hydrochlorothiazide, and amitriptyline. She denied taking over-the-counter medications or supplements. The physical exam on admission revealed a thin woman, afebrile, with a waxing and waning mental status. Blood pressure was 157/75 with a heart rate of 69 beats per minute. Physical examination was notable for absence of jugular venous distention and cardiac rub, presence of mildly decreased breath sounds at the lung bases, and a soft systolic murmur at the right upper sternal border. The abdomen was mildly tender in the hypogastrium. There was no costovertebral tenderness, edema, or rash. She did have asterixis. The Foley catheter bag had a scant amount of dark-brown urine. Laboratory studies were notable for normocytic anemia with a hemoglobin level of 10.6 g/dL, and normal leukocyte and platelet counts. Blood urea nitrogen (BUN) was 157 mg/dL and creatinine 14.5 mg/dL. Serum creatinine levels 3 months prior ranged from 1.3 to 1. 5 mg/dL. Hospital laboratory urinalysis and microscopic exam showed 3+ blood, large protein, and 14 white blood cells per high power field, respectively. Urine sediment revealed numerous red blood cells and red blood cell casts. Hepatitis panel, anti–nuclear antibody (ANA), rapid plasma reagin (RPR), serum and protein electrophoresis, C3 and C4 complement, and p-ANCA (perinuclear-ANCA) were all negative or normal. c-ANCA and proteinase-3 (PR-3) antibody titers were elevated at 1:80 (normal, <1:40) and 64 IU/mL (normal, <6), respectively. Anti-GBM Qanta Lite ELISA detection kit (Quanta Lite ™ GBM, Inova Diagnostics, San Diego, CA) was negative on three separate occasions. A blood sample was also sent to the Mayo Clinic (Rochester, MN) for anti-GBM antibody testing and did not show any circulating anti-GBM antibodies. Renal ultrasound revealed normal-size kidneys with bilateral increased echogenicity. A CT scan of the chest showed two masses in the right lung, as well as several smaller nodules bilaterally, but no findings were suggestive of pulmonary hemorrhage. Over the ensuing 24 h, she remained anuric. Hemodialysis was initiated. She underwent both lung and renal biopsies and was empirically started on high-dose intravenous steroids. Subsequently, transbronchial lung biopsy showed necrotizing granulomatous lesions (Fig. 1).

Light microscopy of the renal biopsy specimen revealed a typical pattern of extracapillary proliferation with crescents and fibrinoid necrosis of the vessel walls (Fig. 2). Endocapillary or mesangial

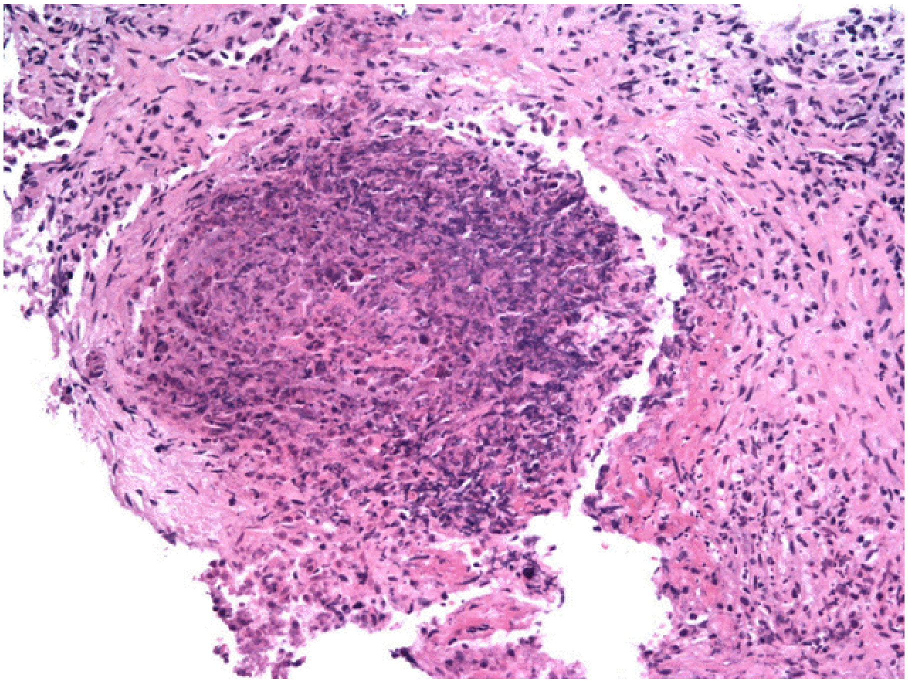

**FIGURE 1.** Wedge lung biopsy. Chronic inflammation and necrotic debris, consistent with a necrotizing granulomatous process (H&E, original magnification ×400).

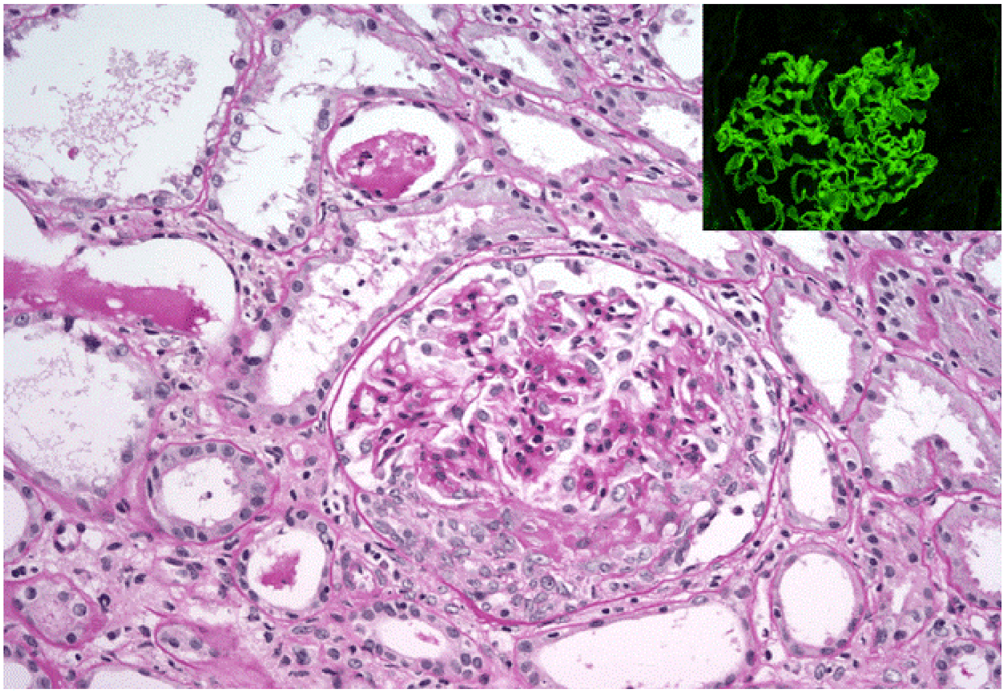

**FIGURE 2.** Renal biopsy**.** Necrotizing and crescentic lesions with marked destruction of the glomerular tuft with fibrin deposition were seen in 15/19 glomeruli. Mild expansion of the interstitium was also present with sparse mononuclear inflammatory infiltrates. Some of the tubules were dilated with loss of nuclei, consistent with a degree of acute tubular necrosis. Direct immunofluorescence of nonsclerotic glomeruli showed bright linear staining for IgG and light chains (inset). Half of them also showed 3+ patchy staining for C3. 1-2 + patchy mesangial staining for IgM and IgA was also seen. There was no evidence of C4 or C1q deposition. (PAS stain; original magnification ×400).

proliferation was not reported. Immunofluorescence revealed patchy C4 and intense linear IgG staining. There was no evidence for diabetic nephropathy. Electron microscopy was remarkable for absence of any subepithelial or subendothelial deposits or fibrils. Given these findings, the patient was given a diagnosis of WG with concurrent anti-GBM disease. She was started on oral cyclophosphamide 75 mg/day. Once immunofluorescence of the biopsy specimen was available and suggested concurrent anti-GBM disease, daily plasmapheresis was initiated. However, since she did not have evidence of pulmonary hemorrhage and her serum anti-GBM antibodies were not detected on several occasions, the treating physicians' decision was to discontinue plasmapharesis after four sessions. Several weeks later, she was discharged to home on hemodialysis and oral cyclophosphamide, which she continued for 3 months. She was then switched to azathioprine. Two years later, she remains dialysis dependent.

## DISCUSSION

The differential diagnosis of a pulmonary-renal syndrome includes immune-complex disorders, such as systemic lupus erythematosus and postinfectious glomerulonephritis, and the pauci-immune vasculitides, such as WG, microscopic polyangitis, Churg-Strauss syndrome, Goodpasture's syndrome, and anti-GBM disease. The diagnosis is supported by both clinical and laboratory findings. However, a lung or renal biopsy may not only add critical information, but may also provide the most specific information.

The sensitivity of both c-ANCA and PR-3 is slightly greater than 70%, while the specificity is 99% for the diagnosis of WG in the presence of a rapidly progressive glomerulonephritis[7]. However, there is an approximate 10% rate of false-negative results depending on the severity of the disease.

The gold standard for diagnosis of anti-GBM disease is tissue biopsy with immunofluorescence showing strong linear staining for IgG. In patients with biopsy-proven anti-GBM disease, the sensitivity of ELISA testing for anti-GBM varies with the specific assay kit used and ranges from 63% to nearly 100%, depending on whether or not the kit utilizes purified antigen. When ELISA is confirmed with Western blot, specificity nears 100%[2,8]. One of the specific ELISA tests used in our case was shown by Jaskowski et al.[8] to have 100% sensitivity. However, it has been observed that a minority of individuals, like our patient, may test negative for circulating anti-GBM antibodies, while immunofluorescence microscopy of the biopsy specimen confirms linear staining for IgG along the GBM[9]. Salama et al., based on their experience in a U.K. reference laboratory, estimated the occurrence of seronegative anti-GBM disease to be 2–3%[10,11].

It is debatable whether ANCA is nonpathogenic or participates in the complex interplay of neutrophils, macrophages, lymphocytes, cytokines, and endothelial cells that lead to necrosis and crescent formation in the kidneys and noncaseating granulomas in the lungs. However, it does appear clear that anti-GBM antibodies are pathogenic. These antibodies form against several epitopes on the noncollagenous-1 (NC-1) domain of the alpha-3 chain of type IV collagen, termed the Goodpasture antigen[12,13,14,15]. These antibodies are usually of the IgG type, although IgA has also been reported[16,17,18].

There is a well-recognized overlap in detectible circulating ANCA and anti-GBM antibodies. When looking at the pauci-immune complex vasculitides, such as WG and anti-GBM disease, it is unclear whether this phenomenon translates into an overlap of the two diseases or is an epiphenomenon. Many hypotheses involving the interplay of these two distinct diseases have been suggested. There appears to be no structural relationship between c-ANCA and the Goodpasture antigen[19]. However, it is possible that the immune response directed towards ANCA or anti-GBM antibodies, by leading to GBM damage and the exposure of the corresponding antigens, may result in sequential development of anti-GBM disease or ANCA-related vasculitis. Indeed, there are case reports of both ANCA vasculitis developing in established anti-GBM disease, as well as of anti-GBM disease that has occurred after the onset of ANCA vasculitis[20]. Some studies have shown that dual positivity confers a beneficial clinical prognosis over anti-GBM disease, which would support some sort of overlap disease rather than an epiphenomenon[21].

However, these results have not been consistent and other studies have not shown any improvement in outcomes[22,23].

Our report describes a patient with lung biopsy–proven, noncaseating, granulomatous disease without any evidence of linear IgG immune complex deposition in the alveolar basement membrane. Concurrently, the same patient had a renal biopsy–proven, necrotizing, crescentic, glomerulonephritis with intense linear complex staining of the GBM. Given the lung biopsy findings, along with elevated ANCA and PR-3 titers, the combination of lung and renal disease would be consistent with WG. However, the intense linear IgG staining on kidney biopsy in the absence of any evidence of diabetic nephropathy or electron-dense deposits or fibrils suggests anti-GBM disease. What adds to these interesting findings is the absence of circulating anti-GBM antibodies as tested by ELISA on several different occasions.

There are several possible explanations for a negative anti-GBM antibody test in our patient. False-negative results are estimated to occur in 2–15% of reported cases[9,24] and are more common in patients with isolated lung involvement. False-negative results may also occur in patients with low antibody titers and in some renal transplant recipients with Alport's syndrome, who have antibodies directed against the alpha-5(IV) collagen chain[25,26]. Also, since patients with dual ANCA and anti-GBM positivity tend to show lower titers of anti-GBM antibodies than those with anti-GBM disease alone[27], antibody titers may fall below the level of detection by available serological testing. This could potentially be a reason for the absence of detectable circulating anti-GBM antibodies in our case. Negative results can also occur when the linear complex antibodies belong to a class other than IgG, since commercial assays test only for the latter. However, this patient's renal biopsy immunofluorescence specimen tested strongly positive for IgG, thus excluding the latter possibility.

Another possible explanation of cases with absent anti-GBM antibodies on serological testing in the presence of positive tissue staining could be that the linear complex staining is not due to anti-GBM disease. For example, cases of fibrillary glomerulonephritis have been reported that present with crescentic glomerulonephritis and linear IgG deposits[28]. However, those cases revealed evidence of fibrillary glomerulonephritis on electron microscopy, which was not present in our patient. Also, other described cases report renal biopsy evidence of linear IgG staining in the absence of any clinical disease. It could be that this patient's clinical signs and symptoms were entirely due to WG, with linear IgG deposits coexisting without clinical significance.

It is puzzling as to why our patient did not have an elevated anti-GBM titer. It is possible that this was a result of a false-negative test, even though the assay used for detection of anti-GBM antibodies in this case (Quanta Lite GBM ELISA detection kit, Inova Corporation), which uses specific purified alpha-3 (IV) collagen chain antigen, was found by Jaskowski et al. to have good sensitivity and specificity (100 and 92.3%, respectively)[8]. However, even in the absence of circulating anti-GBM antibodies, this patient had biopsy evidence compatible with both anti-GBM disease and WG. Our patient's case is unique among other case reports, as it is the only one in which dual disease was actually demonstrated pathologically.
